# Physiochemical and molecular responses of the diatom *Phaeodactylum tricornutum* to illumination transitions

**DOI:** 10.1186/s13068-023-02352-w

**Published:** 2023-06-16

**Authors:** Wei Ding, Ying Ye, Lihua Yu, Meijing Liu, Jin Liu

**Affiliations:** Laboratory for Algae Biotechnology & Innovation, College of Engineering, Peking University, Beijing, 100871 China

**Keywords:** Illumination transitions, Carbon metabolism reorientation, Photosynthesis, Pigments, Lipids, Transcriptomics

## Abstract

**Background:**

Light is a key regulatory factor for photosynthesis and metabolism of microalgae. The diatom *Phaeodactylum tricornutum* is capable of exhibiting metabolic flexibility in response to light fluctuations. However, the metabolic switching and underlying molecular mechanisms upon illumination transitions remain poorly understood for this industrially relevant marine alga. To address these, the physiochemical and molecular responses of *P. tricornutum* upon high light (HL) and recovery (HLR) were probed.

**Results:**

Upon HL, *P. tricornutum* exhibited quick responses, including decreases in cell division, major light harvesting pigments (e.g., chlorophyll *a*, *β*-carotene, and fucoxanthin), chloroplastidic membrane lipids (e.g., monogalactosyldiacylglycerol, digalactosyldiacylglycerol, and sulfoquinovosyldiacylglycerol), and long-chain polyunsaturated fatty acids (e.g., C20:5), as well as increases in carbohydrates and neutral lipids particularly triacylglycerol. During HLR stage when the stress was removed, these physiochemical phenotypes were generally recovered, indicative of a rapid and reversible changes of *P. tricornutum* to cope with illumination transitions for survival and growth. Through the integrated analysis with time-resolved transcriptomics, we revealed the transcriptional control of photosynthesis and carbon metabolism in *P. tricornutum* responding to HL, which could be reversed more or less during the HLR stage. Furthermore, we highlighted key enzymes involved in carotenoid biosynthesis and lipid metabolism of *P. tricornutum* and identified monooxygenases putatively responsible for catalyzing the ketolation step towards fucoxanthin synthesis from neoxanthin.

**Conclusions:**

The detailed profiling of physiochemical and transcriptional responses of *P. tricornutum* to HL-HLR treatments advances our understanding on the adaption of the alga to illumination transitions and provides new insights into engineering of the alga for improved production of value-added carotenoids and lipids.

**Supplementary Information:**

The online version contains supplementary material available at 10.1186/s13068-023-02352-w.

## Background

Diatoms represent a group of widely distributed unicellular eukaryotic algae that contribute about 40% of the global ocean primary productivity and play an important role in the ocean carbon cycle [1]. Being prominent inhabitants of highly productive marine areas, diatoms are typically autotrophs, harvest and convert solar energy to chemical energy for primary biomass production [2]. Sunlight is the primary source of energy and serves as a crucial regulator for diatoms’ physiology and metabolism. In natural habitats, diatoms are vulnerable to movements caused by the turbulent mixing and currents of the ocean, and thus may undergo exposure to light fluctuations frequently. Through the adjustment of their physiology and biochemical activities, the algae acclimatize themselves to light intensity transitions to perform photoautotrophic growth and metabolism for survival [3]. It has been proposed that diatoms can serve as promising bio-based cell factories due to their robustness of growth and adaptability to environmental changes and ability of synthesizing value-added products [4]. Better understanding of the physiology and biology of algae in response to changing conditions aids in the development of algae platform for synthesizing and accumulating compounds of interest.

*Phaeodactylum tricornutum*, a marine model diatom, has been widely studied in the fields of ecology, biochemistry, molecular biology and biorefinery processes [4, 5]. Furthermore, the alga is known to biosynthesize high-value and broad-market compounds, e.g., fucoxanthin, eicosapentaenoic acid (EPA) and docosahexaenoic acid (DHA), and is considered as a commercially feasible strain with large-scale production potential [1, 6, 7]. During the process of artificial cultivation, the target compounds in *P. tricornutum* can be impacted by culture conditions and thus the conditions should be optimized to enhance production. For example, the percentage of DHA in *P. tricornutum* increased following the increases of light intensity (14–150 μmol photons m^−2^ s^−1^) [8]. Wang et al. [9] adopted a two-stage culture strategy for *P. tricornutum* production: under high light intensity for the algal growth and lipid accumulation, and then transitioned to low light intensity for the accumulation of fucoxanthin and EPA [9]. Thus, the growth and fatty acid composition of *P. tricornutum* vary with culture stage and environmental factors, allowing the alga to acclimatize to dynamic environments. Such “acclimation” responses tend to result in physiological changes and metabolic shifts.

It is well known that diatoms, through a complex system of photoreception and sensory- and metabolic pathways, continuously sense, evaluate and modulate their photosynthetic apparatus to acclimate the changes in the intensity of irradiance [10]. In *P. tricornutum* diadinoxanthin (Ddx) cycle serves as one of the most prominent photoprotective mechanisms [11]. When *P. tricornutum* cells acclimated under low light (e.g., 40 μmol photons m^−2^ s^−1^) were transferred to high light (e.g., 1250 μmol photons m^−2^ s^−1^), the alga showed rapid induction of non-photochemical quenching (NPQ) and ca. 20-fold increase in diatoxanthin (Dtx) level, accompanied by the reduction of fucoxanthin content [12]. Nymark et al. reported that during 48 h following the transition of *P. tricornutum* cultures from low light (35 μmol photons m^−2^ s^−1^) to high light (500 μmol photons m^−2^ s^−1^), the photochemical phase of photosynthesis was impacted while the biochemical phase of photosynthesis, i.e., the Calvin cycle, remained relatively stable [11]. Upon shifting *P. tricornutum* cultures from high light (e.g., 300 μmol photons m^−2^ s^−1^) to low light (e.g., 30 μmol photons m^−2^ s^−1^), it was observed that the carbon metabolism was directed towards the production of phosphoenolpyruvic acid (PEP) and/or pyruvate, photosynthesis was depressed while respiration was increased [13]. Despite the recent studies on photoacclimation of *P. tricornutum*, understanding of the molecular mechanisms underlying the algal response to light transitions remains limited.

To gain a global understanding of the response of *P. tricornutum* to high light and recovery treatments, an integrated analysis of time-resolved physiochemical and transcriptomic changes was conducted. In response to HL, cell division, light harvesting pigments, chloroplastidic membrane lipids and long-chain polyunsaturated fatty acids declined, yet the storage compounds such as carbohydrates and neutral lipids (particularly triacylglycerol) increased. These physiochemical phenotypes were more or less recovered when the high light stress was removed. The phenotypic changes were generally supported by the transcriptomics data. Furthermore, key enzymes involved in carotenoid biosynthesis and lipid metabolism of *P. tricornutum* were highlighted and monooxygenases putatively catalyzing the ketolation step towards fucoxanthin synthesis were proposed.

## Methods

### Algal strain and culture conditions

*P. tricornutum* (CCMP2561) was obtained from the cultivated collection of the Provasoli-Guillard National Center for Culture of Marine Phytoplankton, Bigelow Laboratory for Ocean Sciences. The algal was grown in modified F/2 medium (eightfold N and P) supplemented with 20 g L^−1^ sea salt in 250-mL column (3 cm diameter) photoreactors, which were bubbled with 1.5% CO_2_ enriched air and illuminated with 50 μmol photons m^−2^ s^−1^ at 23 °C. When reaching exponential growth phase, the algal cells were harvested and used as the seeds for a two-stage treatment. At the first stage, the seed cells were inoculated into new column photoreactors with fresh medium at a starting cell density of 1.8 × 10^7^ cells mL^−1^ and cultured under 50 μmol photons m^−2^ s^−1^ (control set, CT) or 300 μmol photons m^−2^ s^−1^ (high light set, HL) for 3 days. At the second stage, the HL-treated cells on day 3, after collected and inoculated into new column photoreactors with fresh medium at a starting cell density of 1.8 × 10^7^ cells mL^−1^, were cultured under continuing HL (HLC) as the control or recovered under illumination of 50 μmol photons m^−2^ s^−1^ (HLR) for another 3 days.

### Measurement of growth and chlorophyll fluorescence parameters

Cell number of algal samples was counted under a light microscope by using a hemocytometer, while algal dry weight was determined gravimetrically using pre-weighted Whatman GF/C filter papers (1.2 μm pore size). Chlorophyll fluorescence parameters of algal samples were determined on a pulse amplitude-modulated fluorometer (Walz, Germany) as described by Li et al. [14].

### Determination of intracellular levels of ROS, carbohydrate and protein

For determination of intracellular ROS levels, algal samples were harvested by centrifuging (3500 g, 5 min), and the cell pellet was washed twice with the 0.5 M phosphate buffered saline (pH 7.0). Then the chemical 2′,7′-dichlorodihydrofluorescein diacetate (DCFH-DA; Beyotime, China) was used as the probe to evaluate the fluorescence intensity caused by ROS, as previously described [15]. The determination of protein and carbohydrate contents in the algal samples followed the procedures described previously [16].

### Immunoblot analysis of photosynthetic proteins

Total protein extracted from the fresh algal samples, after determined by a BCA Protein Assay Kit (Beyotime), was run with loading buffer (30 μg) on a 12% SDS-PAGE gel and subsequently transferred to a PVDF membrane, and immunoblotted with anti-PsbD (Agrisera, Sweden), anti-Cyt b6 (Agrisera), antiLhca2 (Agrisera) antibodies. Anti-Histone H3 (Abcam, USA) was selected as the internal reference. After the incubation with an anti-rabbit IgG antibody (BioXCell, USA), the antigen–antibody complexes on the membrane were visualized by using an enhanced chemiluminescence substrate detection kit (Thermo Fisher Scientific, USA) and captured on a ChemiDoc MP imaging system (Bio-Rad, USA).

### Analysis of pigments and lipids

Algal samples, after collected through centrifugation, were homogenized and extracted fully with a solvent mixture (3 mL) of chloroform–methanol (2:1, *v*/*v*) according to previously described procedures [17]. For phase separation, 0.75% NaCl solution (0.75 mL) was added to the solvent extracts and centrifuged. The bottom chloroform layer containing pigments and lipids was collected and dried under nitrogen gas stream.

For the analysis of pigments, the dried extracts were dissolved in acetone and then separated on a high performance liquid chromatography (HPLC) system equipped with a Waters 2996 photodiode array detector and a Waters Spherisorb column (4.6 × 50 mm; Waters, USA), according to our previously described procedures [18]. The wavelength of 450 nm was employed for recording the pigment peaks. Pigments (carotenoids and chlorophyll *a*) were identified according to the retention time and absorption spectra, and quantified using authentic standards (Sigma, USA).

For the analysis of lipids, the dried extracts were dissolved in chloroform. The chloroform samples were dotted on silica gel 60 thin-layer chromatography (TLC) plates (Merck, Germany) and developed with a mixture of hexane:tertbutylmethyl ether:acetic acid (80: 20: 2, by vol) for neutral lipids separation and with a mixture of chloroform:methanol:acetic acid:water (25: 4: 0.7: 0.3, by vol) for polar lipids separation [19]. Triacylglycerol (TAG) and individual polar lipid classes on the TLC plates, after visualization under iodine vapor, were extracted with chloroform for recovery. Lipids were transesterified with sulfuric acid in methanol and the resulting fatty acid methyl esters (FAMEs) were analyzed by using an Agilent 7890 capillary gas chromatograph equipped with a 5975 C mass spectrometry detector and a HP-88 capillary column (60 m × 0.25 mm) (Agilent Technologies, USA) for quantification according to our procedures described previously [20]. The lipid content was expressed as mass of fatty acids of each lipid class per dry biomass weight.

To visualize lipid droplets (LDs) within algal cells, algal samples were stained by incubating with the fluorescent dye BODIPY 505/515 (Molecular Probes, USA) at concentration of 1 μg mL^−1^ for 10 min at room temperature, followed by the observation under an BX51 fluorescence microscope (Olympus, Japan).

### RNA-Seq for the analysis of differentially expressed genes

Algal samples from various time points (3, 6, 12 and 24 h) of the CT, HL, HLC and HLR treatments were collected for RNA-seq. Total RNA from these samples was extracted using the TRIzol Reagent (Invitrogen, USA) following the manufacturer’s instructions (two biological replicates). After DNase I treatment (TaKaRa, Japan), quality check on an Agilent 2100 Bioanalyzer (Agilent Technologies) and quantification on a NanoDrop 2000C (Thermo Scientific, USA), around 10 μg of total RNA from each sample was used for transcriptome library construction and the following sequencing on an Illumina NovaSeq 6000 sequencing system (Illumina, USA) by Majorbio Biotechnology Co., Ltd (Shanghai, China). The clean reads were aligned to the genome of *P. tricornutum* (http://protists.ensembl.org/Phaeodactylum_tricornutum/Info/Index) with the software TopHat (version 2.0.4). The transcriptome data were deposited in the Gene Expression Omnibus with the accession number SUB12915412. The gene transcriptional abundance was calculated as reads per kilobase of transcript per million mapped reads (RPKM) from gene read counts and gene lengths as defined in the respective gene models. Differentially expressed genes (DEGs) were defined as follow: the average FPKM value of at least one group was no less than 1 and gene expression between the treatment group and control (HL versus CT or HLR versus HLC) had no less than a twofold change with the false discovery rate-adjusted p-value less than 0.01.

### Quantitative real‑time PCR for the validation of RNA‑seq data

The total RNA samples for RNA-seq were reversely transcribed to cDNA by using the PrimeScript™ RT Master Mix (TaKaRa, Japan) following the manufacturer’s instructions. Quantitative real-time PCR (qPCR) was performed according to previously described procedures [20] using a 7500 Fast Real-Time PCR System (Applied Biosystems, USA) with SYBR^®^ Premix Ex Taq^™^ II (TaKaRa, Japan). Genes and primers used for qPCR are listed in Additional file 5: Table S1. The gene transcriptional expression level was normalized using the β-actin gene as the internal control.

## Results and discussion

### Effect of the two-stage high light and recovery treatment on the growth and photosynthetic parameters of *P. tricornutum*

**Fig. 1:**
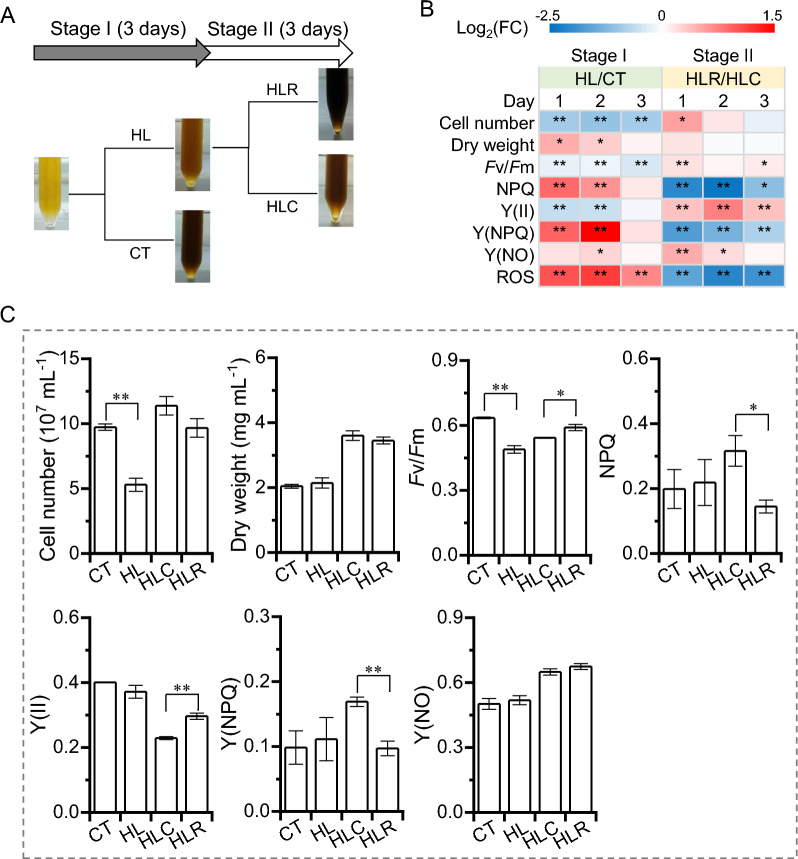
Growth and photosynthetic parameters of *P. tricornutum* during the two-stage treatment. **A** Schematic illustration of the two-stage treatment. **B** Heatmap shows the log2(fold change) values of cell number, dry weight, F*v*/F*m*, NPQ, Y(II), Y(NPQ), Y(NO), and ROS during stage I (HL versus CT) and stage II (HLR versus HLC). **C** Cell number, dry weight, F*v*/F*m*, NPQ, Y(II), Y(NPQ), and Y(NO) of day 3 cultures for CT, HL, HLC and HLR. The significant difference is designated by * (*p* < 0.05) or ** (*p* < 0.01) under Student’s *t*-test

High light is a well-known abiotic stress that has multiplex effects on diatom [21, 22]. Microalgae can coordinate photosynthesis and metabolism to adapt to fluctuations in light [23]. In order to assess the effect of illumination transitions on *P. tricornutum*, a two-stage experiment was performed: stage I, the cultures acclimated under 50 μmol photon m^−2^ s^−1^ were treated with 300 μmol photons m^−2^ s^−1^ (high light set, HL) for 3 days, with the cultures under 50 μmol photon m^−2^ s^−1^ as the control set (CT); stage II, the HL-treated cultures were transferred to 50 μmol photon m^−2^ s^−1^ for recovery (HLR), with the cultures under HL continuing treatment as the control (HLC) for another 3 days (Fig. 1A). During stage I, HL cultures had much lower (e.g., 45.5% on day 3) volumetric cell number than that of CT cultures (Fig. 1B, C). The biomass dry weight, on the other hand, was slightly higher for HL cultures than for CT cultures, particularly on days 1 and 2 (Fig. 1B). These results suggest that HL hinders the cell division of *P. tricornutum* but promotes the per cell weight probably through the increase of cell size and intracellular compounds. This observation is in line with the previous report that high irradiance benefited biomass production of *P. tricornutum* as compared to low light conditions [24]. Be noted that the light intensity for CT was 50 μmol photons m^−2^ s^−1^, which was far below the threshold value (200 μmol photons m^−2^ s^−1^) as stated by Ova Ozcan et al. (2020) [25]. Light becomes a limiting factor for growth when below the threshold [26]. Thus, the algal growth of CT might be severely limited as compared to the optimal light condition. On the other hand, under HL (300 μmol photons m^−2^ s^−1^), although with an impairment in algal photosynthesis potential, the photoinhibition of algal cultures might be not so severe. Combined, the growth (biomass concentration) under HL is lower than that under optimal light but might be still greater than that under CT. Mouget et al. also showed that light intensity affects diatom cellular processes, including motility, sexual reproduction, and cell division [27]. The impairment of cell division has also been reported for *P. tricornutum* that undergoes other stresses such as nutrient limitation [28]. During stage II, the cell number showed only slight difference between HLC and HLR cultures, so did the biomass dry weight (Fig. 1B, C).

*P. tricornutum* is a photosynthetic organism that relies on light to provide energy under photoautotrophic conditions, and its photosynthetic performance may be affected during light transitions. Thus, the photosynthetic parameters were evaluated for *P. tricornutum* cultures. The maximum quantum yield of PSII, measured as F*v*/F*m*, remained lower for HL cultures as compared to CT cultures and reached below 0.5 on day 3, indicative of the occurrence of light stress and an impairment in algal photosynthesis (Fig. 1B, C). NPQ is a switchable mechanism that protects photosynthetic systems from photodamage caused by HL [11]. Clearly, accompanied by the drop in F*v*/F*m*, HL led to a rise in NPQ, which was significant on days 1 and 2 but not on day 3 (Fig. 1B, C). This result firmly supports that the NPQ is a photoprotective measure to dissipate energy by heat emission and protects *P. tricornutum* cells from photodamage when light captured by the light harvesting pigments surpasses the photosynthetic apparatus ability for light utilization in photochemistry [29]. The effective quantum yield of PSII, Y(II), was lower in HL cultures as compared to CT cultures, particularly on days 1 and 2 (Fig. 1B). Y(NPQ) designates the quantum yield of regulated non-photochemical energy loss in PSII, and Y(NO) represents the quantum yield of non-regulated non-photochemical energy loss in PSII [30]. Opposite to the Y(II), Y(NPQ) and Y(NO) were higher for HL cultures than for CT cultures (Fig. 1B). HL also stimulated the level of intracellular reactive oxygen species (ROS; Fig. 1B). These differences reflect that HL causes a photodamage and activates the photoprotection mechanisms of *P. tricornutum*.

During stage II, HLR cultures recovered partially in reproduction as suggested by the greater cell number on day 1 when compared to HLC cultures (Fig. 1B). HLR cultures also showed a recovery of F*v*/F*m*, which is higher than that of HLC cultures (Fig. 1B). As expected, the HLR cultures became relaxed and had lower NPQ and ROS levels than HLC cultures (Fig. 1B, C). This result further demonstrates the function of NPQ in response to light fluctuations and its reversible regulation. Analogously, Lavaud et al. also reported that after treatment of HL and returned to the dark, NPQ showed a rapid relaxation in *P. tricornutum* [31]. As for Y(II), the HLR group was higher than the HLC group (Fig. 1B), indicative of the restoration of the photochemistry of photosynthesis. The changes of Y(NPQ) were in good agreement with the observed tendency of NPQ (Fig. 1B). Remarkably, the HLR group had higher Y(NO) values than the HLC group (Fig. 1B), suggesting that the photodamage caused by HL treatment still existed despite the recovery of photosynthetic system during stage II. These results appear to be evident from the fact that HL exposure and removal induce reversible switches in photosynthesis, during which the photosynthetic performance is impaired and reactivated.

To investigate effect of the two-stage treatment on the protein abundance of photosynthetic apparatus, the immunoblot analysis of whole protein from *P. tricornutum* was performed, using histone H3 as the internal control. D2 protein of PSII (PsbD) is one of the core complex intrinsic proteins that constitute the reaction center of PSII and contribute to the photochemical charge transfer. PsbD protein had a considerably lower abundance under HL as compared to CT (Additional file 1: Fig. S1). Interestingly, when the HL cultures were subjected to recovery during stage II (HLR), the abundance of PsbD protein did not restore and remained at a very low level. Thylakoid membrane cytochrome b6 protein (Cyt b6), a marker for the inter-photosystem electron transport chain, also declined considerably in response to HL stress, yet was restored slightly during the recovery stage (Additional file 1: Fig. S1). LHC of PSI (LHCI), on the other hand, exhibited only slight variations when subjected to HL and HLR (Additional file 1: Fig. S1). Probably, LHCs are more stable than the core proteins and are involved in efficient energy transfer to protect the alga under stress conditions. These results show that HL stress mainly affects the PS II reaction centers rather than the electron transport chain of photosynthesis, and leads to a decrease in the effective photochemical quantum yield of PSII.

### Effect of the two-stage high light and recovery treatment on the pigment profiles of *P. tricornutum*

**Fig. 2:**
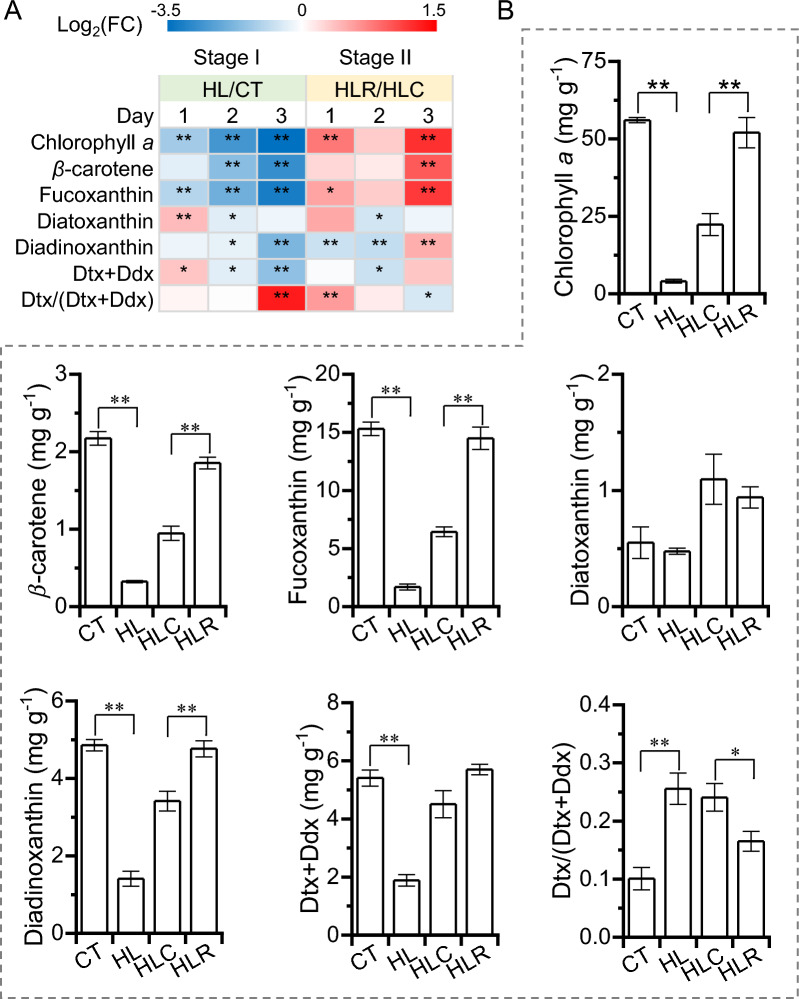
Pigment profiles of *P. tricornutum* during the two-stage treatment. **A** Heatmap shows the log2(fold change) values of β-carotene, fucoxanthin, diatoxanthin (Dtx), diadinoxanthin (Ddx), pool of Dtx and Ddx (Dtx + Ddx), and ratio of Dtx/(Dtx + Ddx). **B** β-carotene, fucoxanthin, diatoxanthin (Dtx), diadinoxanthin (Ddx), pool of Dtx and Ddx (Dtx + Ddx), and ratio of Dtx/(Dtx + Ddx) of day 3 cultures for CT, HL, HLC and HLR. The significant difference is designated by * (*p* < 0.05) or ** (*p* < 0.01) under Student’s *t*-test

*P. tricornutum* performs light harvesting mainly by fucoxanthin chlorophyll protein (FCP) complexes, which contain three light-harvesting pigments including fucoxanthin, chlorophyll *a* (Chl *a*), and chlorophyll *c* (Chl *c*) [32]. During stage I, HL caused a severe decline in the levels of Chl *a* and fucoxanthin, which were ca. 92.8% and 89% lower than CT on day 3, respectively (Fig. 2A, B). The β-carotene level was also greatly less in HL cultures than that in CT cultures (Fig. 2A, B). The significant decrease of Chl a, fucoxanthin and β-carotene after prolonged HL exposure supports that *P. tricornutum* cuts down synthesized light-harvesting pigments as a countermeasure against the substantial HL stress [12]. During stage II, Chl *a*, fucoxanthin and β-carotene recovered gradually for HLR cultures, which were much greater than those for HLC cultures (on day 3) and reached the levels comparable to CT cultures on day 3 (Fig. 2A, B). It is worth mentioning that the levels of Chl *a* and fucoxanthin correlated well when *P. tricornutum* were exposed to different conditions (Additional file 1: Fig. S2). These results suggest that in *P. tricornutum* the major light-harvesting pigments have plasticity in response to light fluctuations.

In diatoms the diadinoxanthin (Ddx) cycle that consists of the interconversion between diadinoxanthin and diatoxanthin (Dtx) plays an important role in photoprotection when exposing to strong illumination [12]. During stage I, diatoxanthin showed an increase responding to HL on day 1 and then declined; diadinoxanthin, on the other hand, decreased gradually to 1.4 mg g^−1^ on day 3 for HL cultures, which was 71% less than that for CT cultures (Fig. 2A, B). The diadinoxanthin and diatoxanthin pool (Ddx + Dtx) also increased on day 1 and then declined during stage I (Fig. 2A). Dtx/(Ddx + Dtx), the de-epoxidation state index (DES), was higher in HL cultures as compared to CT cultures, particularly on day 3 (Fig. 2A, B), indicative of the enhanced conversion of diadinoxanthin to diatoxanthin upon HL to protect the algal cells from photodamage. When transferring to stage II for recovery, HLR cultures remained low in level of diatoxanthin and was comparable to HLC cultures, yet having a diadinoxanthin level lower on days 1 and 2 and higher on day 3 than HLC cultures (Fig. 2A, B). Considering the changes of fucoxanthin and diadinoxanthin, the latter may serve as a precursor of the former, as previously suggested [33]. Seemingly, the light-harvesting pigments in *P. tricornutum* (eg. Chl *a*, fucoxanthin and β-carotene) are sensitive to light transitions and can recover well once the HL stress is relieved, while the photoprotective carotenoids are not.

### Effect of the two-stage high light and recovery treatment on protein, carbohydrates, and lipids of *P. tricornutum*

**Fig. 3:**
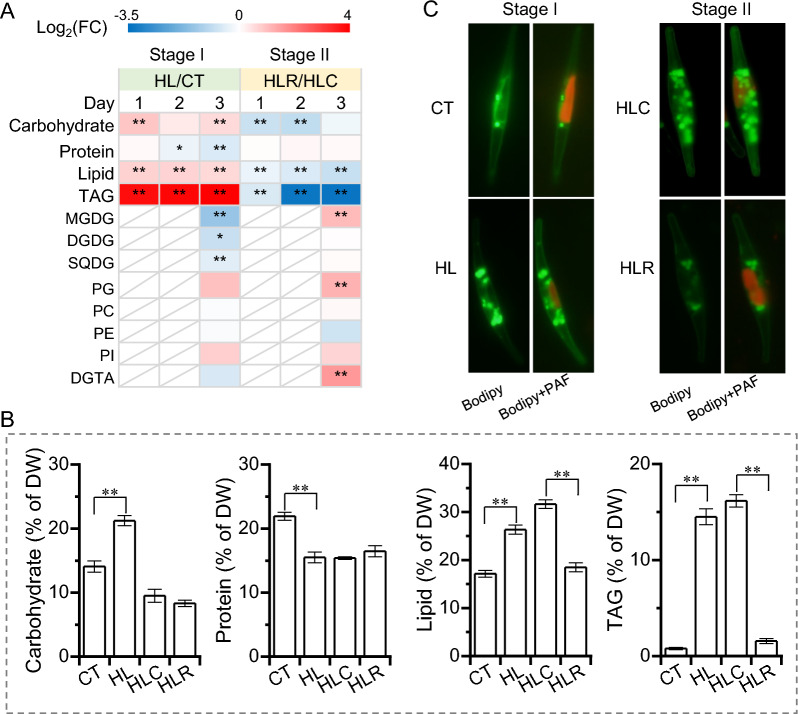
Carbohydrate, protein and lipid profiles of *P. tricornutum* during the two-stage treatment. **A** Heatmap shows the log2(fold change) values of carbohydrate, protein and lipid contents. **B** Carbohydrate, protein and lipid contents of day 3 cultures for CT, HL, HLC and HLR. **C** Microscopic observation of algal cells stained by the fluorescence dye Bodipy. Green fluorescence indicates the Bodipy-bound TAG-filled lipid droplets, while red indicates the plastid autofluorescence (PAF). The significant difference is designated by * (*p* < 0.05) or ** (*p* < 0.01) under Student’s *t*-test

*P. tricornutum* bio-fixes CO_2_ and synthesizes carbohydrates, protein, and lipids as the major compounds within cells. During stage I, the protein content decreased by HL and was 29.24% lower than CT on day 3 (Fig. 3A, B). By contrast, the carbohydrate content was significantly enhanced by the HL treatment, reaching 21.23% of dry weight on day 3 and 50.1% higher than CT (Fig. 3A, B). The lipid content was also greater for HL cultures, which accounted for 26.3% of dry weight on day 3 and was 50% greater than CT cultures (Fig. 3A, B). Triacylglycerol (TAG), on the other hand, was even more considerably promoted by HL; its content on day 3 represented 14.5% of dry weight for HL cultures and was 18.3-fold greater than CT cultures (Fig. 3A, B). The HL-induced strong TAG accumulation was also evidenced by the occurrence of much more TAG-filled lipid droplets, which were obvious under microscopic observation of the algal cells stained with BODIPY, a specific fluorescence dye binding to neutral lipids (Fig. 3C). During stage II for recover, HLR cultures showed a decline in carbohydrate and lipid (including TAG) levels, which were significantly lower than that in HLC cultures (Fig. 3A–C). Protein level, on the other hand, had little difference between HLR and HLC cultures (Fig. 3A). These results suggest that HL benefits the accumulation of storage compounds such as carbohydrates and neutral lipids, which can be quickly reversed when HL stress is relieved.

In addition to the neutral lipid TAG, *P. tricornutum* contains many polar lipids that are important building blocks of membranes, including monogalactosyldiacylglycerol (MGDG), digalactosyldiacylglycerol (DGDG), sulfoquinovosyldiacylglycerol (SQDG), phosphatidylglycerol (PG), diacylglyceryl-hydroxymethyl-N, N, N-trimethyl-b-alanine (DGTA), phosphatidylcholine (PC) and phosphatidylethanolamine (PE), and phosphatidylinositide (PI) [34]. MGDG, DGDG and SQDG, the most abundant plastid membrane lipid classes in *P. tricornutum*, declined 62%, 40.8%, and 24.5%, respectively, when exposed to HL for 3 days (Fig. 3A), consistent with the decrease of major photosynthetic pigments (Fig. 2). DGTA showed a slight decrease, PC and PE had almost no change, while PG and PI increased mildly when algal cells were treated by HL (Fig. 3A). During stage II, of the major plastid membrane lipids, only MGDG of HLR cultures recovered and was considerably higher than that of HLC cultures (Fig. 3A). These results indicate that HL triggers remodeling of membrane lipids, particularly the plastid lipids, which likely provide precursors for TAG assembly, similar to the effect of other stresses such as nitrogen deprivation [35]. These membrane lipids, nevertheless, only recover partially when the HL stress is removed.

**Fig. 4:**
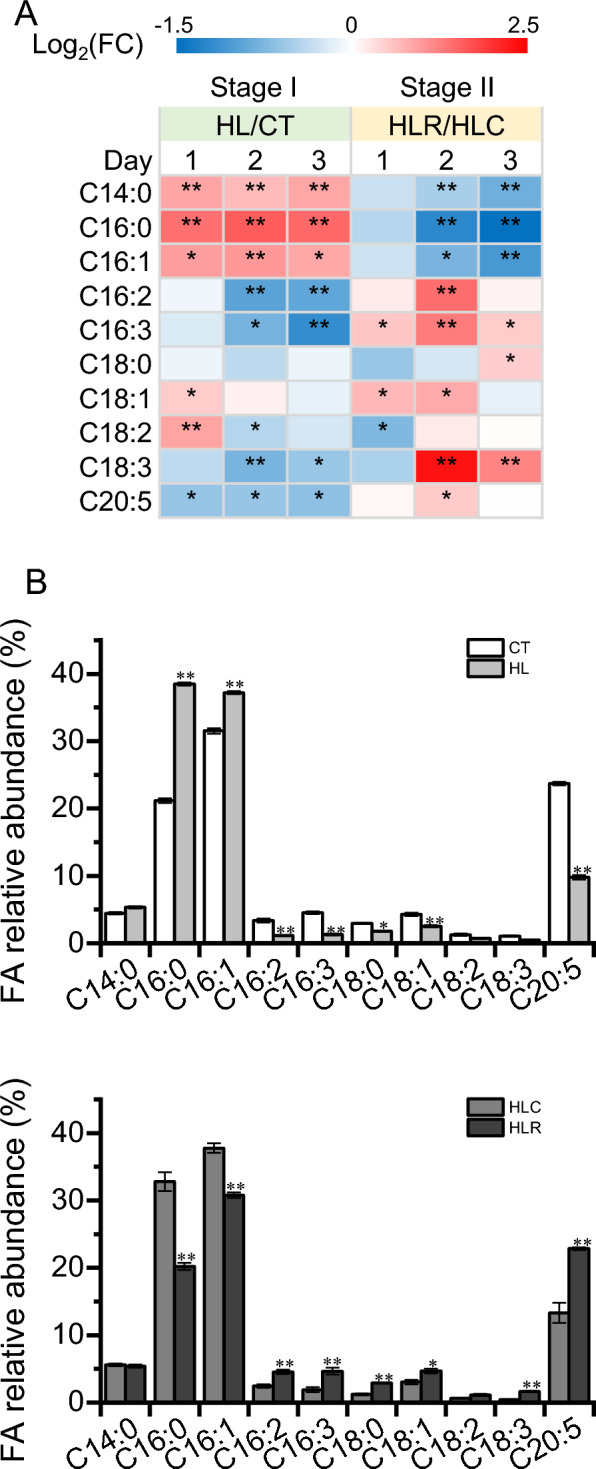
Fatty acid profiles of *P. tricornutum* during the two-stage treatment. **A** Heatmap shows the log2(fold change) values of individual fatty acids. **B** Fatty acid relative abundance in lipids of day 3 cultures for CT, HL, HLC and HLR. FA, fatty acid. The significant difference is designated by * (*p* < 0.05) or ** (*p* < 0.01) under Student’s t-test

The lipids of *P. tricornutum* consist of a serial of fatty acids, with C16:0, C16:1 and C20:5 being the major ones [36]. The fatty acid composition of *P. tricornutum* is subject to changes dependent on the culture conditions [8]. To see how the fatty acids of *P. tricornutum* respond to the two-stage HL and recovery treatment, they were quantified by GC–MS. Clearly, C16:0, C16:1 and C20:5 represented the major fatty acids under all tested culture conditions (Fig. 4 and Additional file 1: Fig. S3). The levels of C14:0, C16:0, and C16:1 (based on the dry weight) increased considerably upon HL and then recovered when the HL stress was relieved (Fig. 4A). The polyunsaturated fatty acids such as C16:2, C16:3, C18:3 and C20:5, on the other hand, declined following the HL treatment and recovered upon the removal of stress (Fig. 4). In this context, *P. tricornutum* favors to synthesize saturated/monounsaturated fatty acids at the expense of polyunsaturated ones under HL stress conditions. As polyunsaturated fatty acids are enriched in the plastid membrane lipids particularly MGDG [34], they showed a positive correlation (Figs. 3 and 4).

### Global gene expression changes at the transcriptional level to the two-stage high light and recovery treatment

To understand molecular mechanisms underlying the physiological and biochemical responses of *P. tricornutum* to the two-stage high light and recovery treatment, a comparative transcriptomics analysis was performed, using samples from 4 time points (3, 6, 12 and 24 h) for stage I and stage II, respectively. A total of 32 transcriptomes were generated. According to the principal component analysis (PCA), the samples showed distinct clusters and had high repeatability among each two biological replicates (Additional file 1: Fig. S4A). In total, 11,635 genes were mapped to the genome of *P. tricornutum* (see Additional file 2: Data S1). During stage I, there were 4233, 3033, 4120 and 5006 differentially expressed genes (DEGs) for 3, 6, 12, and 24 of HL versus CT, respectively (Additional file 1: Fig. S4C). There were slightly more down-regulated DEGs than up-regulated DEGs for HL versus CT (Additional file 1: Fig. S4C). During stage II, the number of DEGs was considerably lower as compared to that of stage I, and there were slightly less down-regulated DEGs than up-regulated DEGs for HLR versus HLC (Additional file 1: Fig. S4B, C). Moreover, we focused on the analysis of gene expression dynamics of certain pathways such as photosynthesis and CO_2_ fixation, central carbon metabolism, carotenoid biosynthesis, and lipid metabolism (Additional file 3: Data S2), which were detailed in the following sections.

### ***Regulation of photosynthesis and CO***_***2***_*** fixation***

**Fig. 5:**
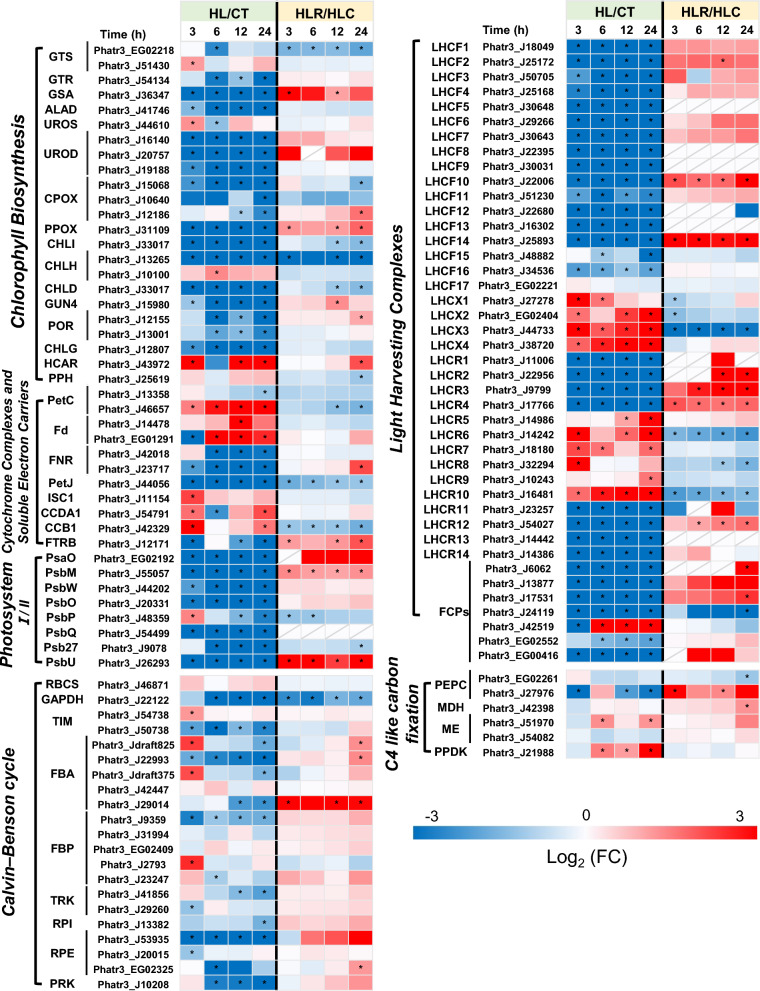
Photosynthesis and CO_2_ fixation pathways in *P. tricornutum* with the heatmap showing the gene expression changes during the two-stage treatment. *GTS* glutamyl-tRNA synthetase, *GTR* glutamyl-tRNA reductase, *GSA* glutamate-semialdehyde aminotransferase, *ALAD* amino levulinic acid dehydratase, *UROS* uroporphyrinogen III synthase, *UROD* uroporphyrinogen III decarboxylase, *CPOX* coproporphyrinogen-III oxidase, *PPOX* protoporphyrinogen IX oxidase, *CHL* Mg-chelatase, GUN4 tetrapyrrole binding protein, *POR* light-dependent protochlorophyllide oxidoreductase, *CHLG* chlorophyll synthetase, *HCAR* 7-hydroxymethyl chlorophyll a reductase, *PPH* pheophytinase, *PetC* cytochrome b6-f complex iron–sulfur subunit, *Fd* ferredoxin, *FNR* ferredoxin NADP reductase, *ISC1* Fe–S cluster assembly factor, *CCDA1* cyt c-Type biogenesis factor, *CCB1* cyt c-Type biogenesis factor, *FTRB* ferredoxin-thioredoxin reductase, *PsaO* photosystem I subunit PsaO, *PsbM* photosystem II reaction center M protein, *PsbW* photosystem II PsbW protein, *PsbO* photosystem II oxygen-evolving enhancer protein 1, *PsbP* photosystem II oxygen-evolving enhancer protein 2, *PsbQ* photosystem II oxygen-evolving enhancer protein 3, *Psb27* Photosystem II subunit 27, *PsbU* photosystem II extrinsic protein, *LHC* light harvest complex protein, *FCP* fucoxanthin chlorophyll a/c protein, *RBCS* ribulose-1,5-bisphosphate carboxylase small subunit, *GAPDH* glyceraldehyde 3-phosphate dehydrogenase, *TIM* triosephosphate isomerase, *FBA* fructose-bisphosphate aldolase, *FBP* fructose-1,6-bisphosphatase, *TRK* transketolase, *RPI* ribose 5-phosphate isomerase, *RPE* ribulose-phosphate 3-epimerase, *PRK* phosphoribulokinase, *PEPC* phosphoenolpyruvate carboxylase, *MDH* malate dehydrogenase, *ME* malic enzyme, *PPDK* pyruvate phosphate dikinase

In response to HL treatment during stage I, the majority of genes involved chlorophyll biosynthesis were considerably down-regulated (Fig. 5). This trend also occurred for genes encoding cytochrome complexes and soluble electron carriers and photosystem I/II components. During stage II when the HL stress was removed, while most of the chlorophyll biosynthetic genes showed slight changes, only several ones increased and recovered their transcriptional levels; interestingly, GTS (Phatr3_EG02218), CHLD (Phatr3_J33017) and CHLH (Phatr3_J13265) remained down-regulated (Fig. 5). Four genes relate to cytochrome complexes and soluble electron carriers, showed a reversible change during the two-stage HL and recovery treatment. Unexpectedly, PetJ (Phatr3_J44056) expression was down-regulated during both stages (Fig. 5). The genes involved in photosystem I/II also exhibited reversible changes upon HL stress and removal. Light harvesting complex (LHC) proteins, a large family of proteins with important function in the photosystem, perform diverse roles including light harvesting, photoprotection and photosystem regulation [37]. In diatoms, LHC proteins consist of three main groups: the major fucoxanthin Chl a/c binding proteins LHCFs, the stress-responsive LI818/LHCSR-like LHCXs, and the red algal-like LHCRs [38]. According to a recent evolutionary analysis, LHCR proteins can be divided into two subclasses: LHCRI (LHCR1-4, 11–14) and LHCRII (LHCR5-10) [3]. Obviously, LHC genes showed differential changes in their transcriptional level upon HL treatment: LHCF and LHCRI genes were down-regulated, while LHCX and LHCRII genes were up-regulated (Fig. 5). Many unclassified LHC genes also saw down-regulation at the onset of HL. According to the previous study, LHCX proteins provide photoprotection via the thermal dissipation of absorbed light and a reduction in the functional absorption cross section of photosphere II [39]. This regulation of the functional absorption cross section can be tuned by altered LHCX proteins expression in response to environmental conditions [40, 41]. Furthermore, LHCRII showed the same trend as LHCX, suggesting that these proteins and LHCX probably have similar regulatory functions. However, this assumption needs to be tested further. When the HL stress was removed, many LHC genes showed a recovery at their transcriptional levels, yet to different extents (Fig. 5).

For the genes involved in the Calvin–Benson cycle responsible for photosynthetic fixation of CO_2_, many showed a transcriptional down-regulation in response to HL (Fig. 5). Notably, several genes were up-regulated transiently, including TIM (Phatr3_J54738), FBA (Phatr3_Jdraft825, Phatr3_Jdraft375) and FBP (Phatr3_J2793), suggesting that HL can stimulate certain carbon fixation related genes. For the C4-like pathway, two key enzymes ME and PPDK were up-regulated, suggesting that the C4 pathway is somewhat enhanced to compensate for the suppressed CO_2_ fixation of Calvin–Benson cycle, thus maintaining the biomass production of HL cultures (Fig. 1). When the HL stress was removed, the genes involved in the Calvin–Benson cycle and C4-like pathway generally recovered (Fig. 5).

### Regulation of center carbon metabolism

**Fig. 6:**
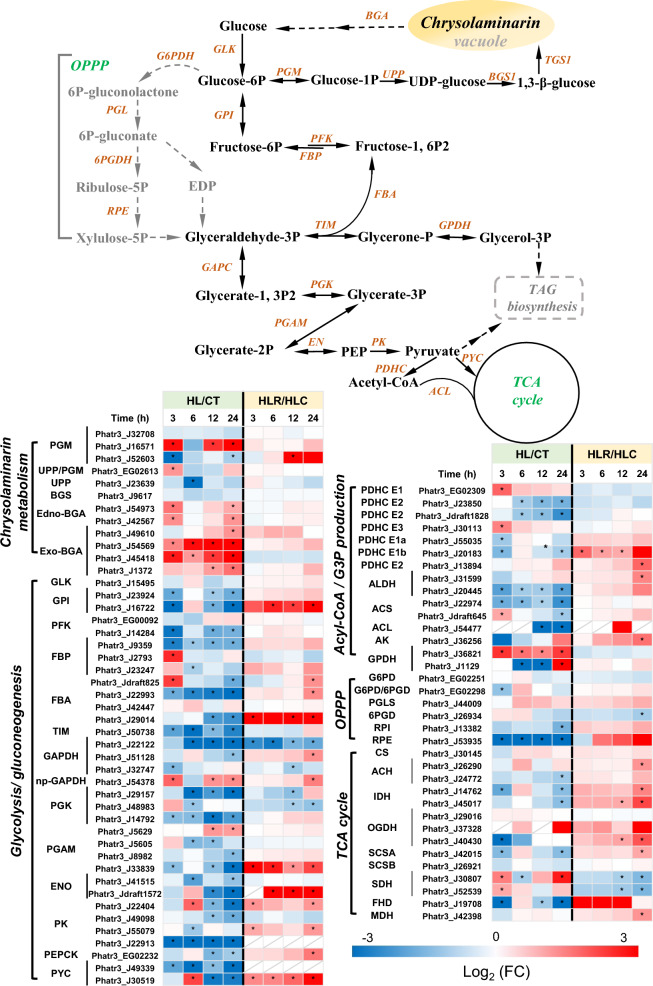
Central carbon metabolism in *P. tricornutum* with the heatmap showing the gene expression changes during the two-stage treatment. *PGM* phosphoglucomutase, *UPP/UDP-glucose* pyrophosphorylase, *BGS* 1,3-beta-glucan synthase, *BGA* β-glucanase, *GLK* glucokinase, *GPI* glucose-6-phosphate isomerase, *PFK* 6-phosphofructokinase, *FBP* fructose-1,6-bisphosphatase, *TIM* triosephosphate isomerase, *GAPDH* glyceraldehyde 3-phosphate dehydrogenase (NAD), *np-GAPDH* glyceraldehyde 3-phosphate dehydrogenase (nonphosphorylating), *PGK* phosphoglycerate kinase, *PGAM* phosphoglycerate mutase, *ENO* enolase, *PK* pyruvate kinase, PEPCK phosphoenolpyruvate carboxykinase, *PYC* pyruvate carboxylase, *PDHC* pyruvate dehydrogenase complex, *ALDH* aldehyde dehydrogenase, *ACS* acetyl-CoA synthetase, *ACL* ATP-citrate lyase, *AK* acetate kinase, GPDH glycerol-3-phosphate dehydrogenase, *G6PD* Glucose-6-phosphate 1-dehydrogenase, *PGLS* 6-phosphogluconolactonase, *6PGD* 6-phosphogluconate dehydrogenase, *RPI* ribose 5-phosphate isomerase, *RPE* ribulose-phosphate 3-epimerase, *CS* citrate synthase, *ACH* aconitate hydratase, *IDH* isocitrate dehydrogenase, *OGDH* 2-oxoglutarate dehydrogenase, *SCS* succinyl-CoA synthetase, *SDH* succinate dehydrogenase, *FHD* fumarate hydratase, *MDH* malate dehydrogenase

Center carbon metabolism plays a crucial role in carbon distribution primarily toward the generation of dominating macromolecule (i.e., carbohydrates, proteins, and lipids), and functions importantly in response to environmental shifts [42]. In β-1,3-glucan biosynthetic pathway, phosphoglucomutase (PGM), catalyzing the committed step of chrysolaminarin biosynthesis, was up-regulated by HL treatment; other ones, on the other hand, showed little change (Fig. 6). Interestingly, in β-1,3-glucan degradation pathway, two enzymes endo- and exo-β-glucanases (endo-BGA, exo-BGA) that catalyze chrysolaminarin breakdown to glucose molecules, were also substantially up-regulated upon HL (Fig. 6). These results imply that the enhancement of carbohydrate under HL conditions is not due to accumulation of chrysolaminarin, but rather some intermediates in glycolysis. When the HL stress was removed, the expression level of chrysolaminarin metabolism genes showed little change, while PGM (Phatr3_J52603) was up-regulated. Glycolysis is a fundamental pathway, as it supplies substrates for energy metabolism within cells. Overall, the genes involved in glycolysis/gluconeogenesis and oxidative pentose phosphate pathway were down-regulated by HL; the exception was observed for GAPDH (Phatr3_J54378) and PGAM (Phatr3_J5629), which were up-regulated in the HL group (Fig. 6). With regard to the genes involved in acyl-CoA and G3P production, besides the up-regulation of PHDC, AK and GPDH at certain time points, others were down-regulated. Furthermore, down-regulation was observed for genes involved in the TCA cycle upon HL with the exception of OGDH (Phatr3_J37328) and GDH (Phatr3_J30807) that were up-regulated.

Upon removal of the HL stress, a majority of genes involved in glycolysis/gluconeogenesis showed a recovery in their transcriptional expression including those encoding enzymes for catalyzing reversible reaction, such as GPI (Phatr3_J16722), three FBAs and two ENOs. As for the enzymes in irreversible steps of glycolysis, glucokinase (GLK), phosphofructokinase (PFK), and pyruvate kinase (PK), only PK was up-regulated. The oxidative pentose phosphate pathway genes showed slight recovery, except for 6PGDH. In acyl-CoA and G3P production processes, most of the DEGs were up-regulated except GPDH. Most noticeably, two genes encoding proteins PYC and ACL were significant up-regulated, probably for providing more pyruvate and acyl-CoA towards TCA cycle. Based on these results, cell metabolism is oriented to recycle components and to use the energy and reducing power through central carbon metabolism and TCA cycle [43, 44]. Furthermore, TCA serves as a recycling of the carbon backbones derived from proteins and amino acids [45]. Consistently, the genes related to TCA cycle showed varying degrees of up-regulation, despite SDH that was down-regulated. These results suggest HL is detrimental to glycolysis/gluconeogenesis and partial recovery can be achieved once the stress is removed. However, there are also several potential compensatory pathways, such as TCA cycle, which is employed primarily to generate energy and reducing power, and oxidative pentose phosphate pathway, which is activated to maintain primary metabolism [28, 46].

### Regulation of carotenogenic pathways and proposed missing enzymes for fucoxanthin synthesis

**Fig. 7:**
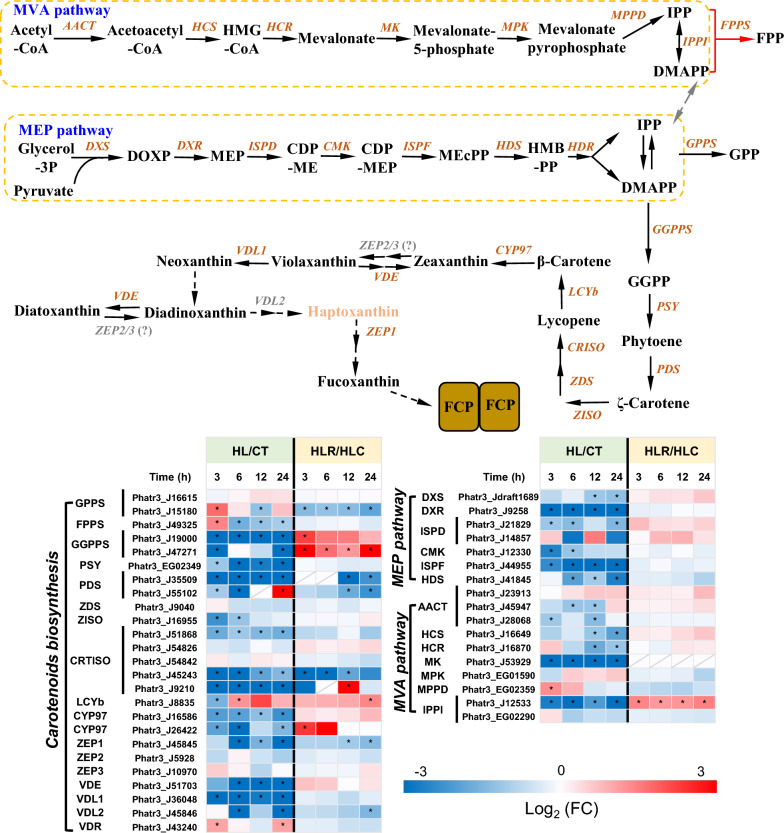
Carotenogenesis in *P. tricornutum* with the heatmap showing the gene expression changes during the two-stage treatment. *DXS* 1-deoxy-D-xylulose 5-phosphate synthase, *DXR* 1-deoxy-D-xylulose 5-phosphate reductoisomerase, *ISPD* 2-C-methyl-D-erythritol 4-phosphate cytidylyltransferase, *CMK* 4-diphosphocytidyl-2-C-methyl-D-erythritol kinase, *ISPF* 2-C-methyl-D-erythritol 2,4-cyclodiphosphate synthase, *HDS* 4-hydroxy-3-methylbut-2-en-1-yl diphosphate synthase, AACT acetoacetyl-CoA thiolase, HCS hydroxymethylglutaryl-CoA synthase, *HCR* HMG-CoA reductase, *MK* mevalonate-5-kinase, *MPK* phosphomevalonate kinase, *MPPD* mevalonate-5-pyrophosphate decarboxylase, *IPPI* Isopentenyl-diphosphate Delta-isomerase, *GPPS* geranyl diphosphate synthase, *FPPS* farnesyl diphosphate synthase, *GGPPS* geranylgeranyl diphosphate synthase, *PSY* phytoene synthase, *PDS* phytoene desaturase, *ZDS* Zeta-carotene desaturase, *ZISO* Zeta-carotene isomerase, *CRTISO* carotenoid isomerase, *LCYb* lycopene beta cyclase, *CYP97A* cytochrome P450 beta hydroxylase, *ZEP* zeaxanthin epoxidase, *VDE* violaxanthin de-epoxidase, *VDL* violaxanthin de-epoxidase like, *VDR* violaxanthin de-epoxidase-related

Isopentenyl diphosphate (IPP) and dimethylallyl diphosphate (DMAPP) serve as the primary precursors for carotenoid biosynthesis. Similar to plants, *P. tricornutum* harbors two pathways towards IPP/DMAPP production, the 2-C-methylerythritol 4-phosphate (MEP) pathway and mevalonate (MVA) pathway [47], differing from green algae and *Nannochloropsis* species that lack complete MVA pathway [48–50]. Many MEP and MVA genes in *P. tricornutum* were down-regulated when exposed to HL and then recovered slightly when the HL stress was removed (Fig. 7). It has been reported previously that a down-regulation of MEP genes occurs in *P. tricornutum* under irradiance conditions of 500 μmol m^−2^ s^−1^ [11]. It is worth mentioning that IPP delta-isomerase (IPPI, Phatr3_J12533), down-regulated severely upon HL, was up-regulated considerably upon HL stress removal (Fig. 7) and correlated well with the changing pattern of fucoxanthin (Fig. 2A), indicative of an important role of this enzyme in IPP/DMAPP production for fucoxanthin synthesis.

Condensation of one DMAPP and three IPP molecules, catalyzed by GGPP synthase (GGPPS), leads to the formation of GGPP. Phytoene synthase (PSY) mediates the condensation of two GGPP molecules to produce phytoene, which is catalyzed by phytoene desaturase (PDS), ζ-carotene isomerase (ZISO), ζ-carotene desaturase (ZDS), carotenoid isomerase (CRTISO) and lycopene β-cyclase (LCYB) in succession to form β-carotene. These genes were generally down-regulated by HL treatment yet only several ones were recovered in response to the stress removal, e.g., GGPPS (Fig. 7). In this context, GGPPS is likely a crucial enzyme involved in carotenoid biosynthesis. Nymark et al. [11] also reported the down-regulation of GGPPS upon high light intensity in *P. tricornutum* and the reduction of Chl *a* and fucoxanthin [11]. Through hydroxylation on both ends, β-carotene is converted to the xanthophyll zeaxanthin. This reaction is mediated by the heme-containing cytochrome P450 enzymes (CYP97) rather than the non-heme di-iron type of β-carotenoid hydroxylase (CHYb) in *P. tricornutum* [51]. Zeaxanthin epoxidase (ZEP) and violaxanthin de-epoxidase (VDE) catalyze the interconversion between zeaxanthin and violaxanthin; the latter can also be converted to neoxanthin by the action of a violaxanthin de-epoxidase like (VDL) in *P. tricornutum* [52], rather than by a neoxanthin synthase in green algae [48]. In addition to the violaxanthin cycle, there is a diadinoxanthin cycle present in *P. tricornutum*, probably involving ZEP and VDE as well for interconversion between diadinoxanthin and diatoxanthin. While ZEP2/3 may be involved in converting zeaxanthin to violaxanthin and diadinoxanthin to diatoxanthin, ZEP1 functions in converting haptoxanthin to phaneroxanthin [33]. The above-mentioned genes involved in the fucoxanthin synthesis from β-carotene were down-regulated by HL, yet only a couple of ones were up-regulated when the HL stress was removed (Fig. 7).

Fucoxanthin contains a keto group and an acetyl group compared to neoxanthin, indicative of the involvement of enzymes for ketolation and acetylation that remain to be disclosed [1]. It is speculated that the ketolation step may be catalyzed by certain monooxygenases. Four out of eleven putative monooxygenases, namely, Phatr3_J8324, Phatr3_J38325, Phatr3_J47925 and Phatr3_J44417, showed a down-regulation upon HL treatment (Additional file 4: Data S3), correlated with the expression pattern of most carotenoid biosynthesis enzymes (Fig. 7) and fucoxanthin changes (Fig. 2). Upon HL stress removal, Phatr3_J38325 and Phatr3_J47925 were up-regulated (Additional file 4: Data S3). In this context, Phatr3_J38325 and Phatr3_J47925 are probably involved in the ketolation step towards fucoxanthin synthesis. Nevertheless, further experiments (e.g., knockout or knockdown) are needed for the functional validation of these genes.

### Regulation of lipid metabolism

**Fig. 8:**
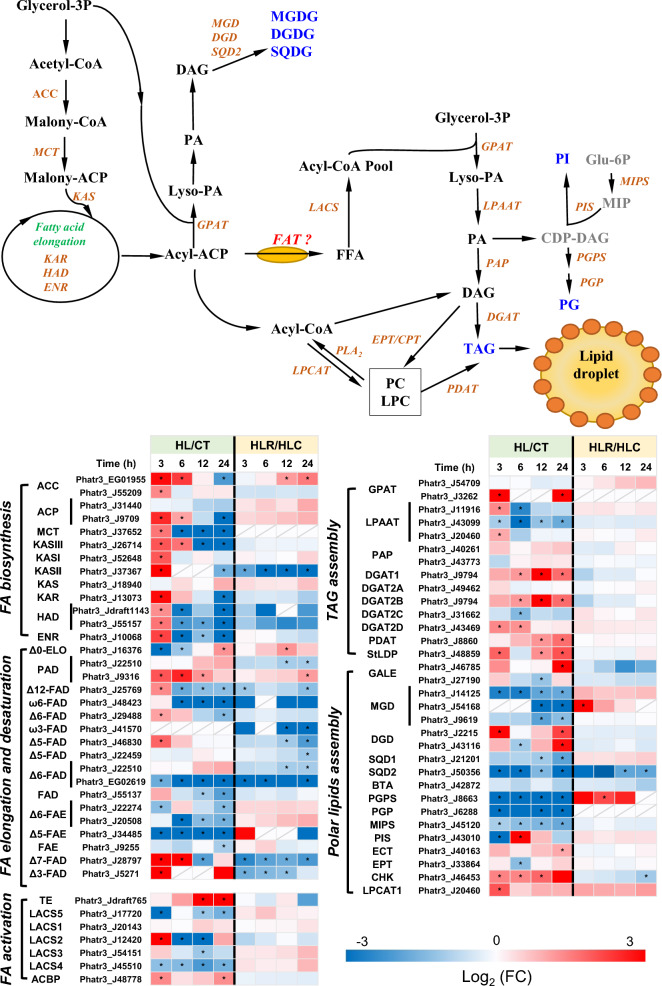
Lipid biosynthesis and remodeling pathways in *P. tricornutum* with the heatmap showing the gene expression changes during the two-stage treatment. *ACC* acetyl-CoA carboxylase, *ACP* acyl carrier protein, *MCT* malonyl-CoA: acyl carrier protein transacylase, *KAS* 3-ketoacyl-ACP synthase, *KAR* 3-ketoacyl-ACP reductase, *HAD* 3-ketoacyl-ACP dehydratase, *ENR* enoyl-ACP reductase, *TE *Acyl-CoA thioesterase, *LACS* long-chain acyl-CoA synthetase, *ACBP* Acyl-CoA-binding protein, *ELO* fatty acid elongase, *PAD* palmitoyl-ACP delta-9-desaturase, *FAD* fatty acid desaturase, *FAE* fatty acid elongase, *GPAT* glycerol-3-phosphate acyltransferase, *LPAAT* lysophospholipid acyltransferases, *PAP* phosphatidate phosphatase, *DGAT* diacylglycerol acyltransferase, *PDAT* phospholipid: diacylglycerol acyltransferase, *StLDP* stramenopile-type lipid droplet protein, *GALE* UDP-galactose 4-epimerase, *MGD* monogalactosyldiacylglycerol synthase, *DGD* digalactosyldiacylglycerol synthase, *SQD* sulfoquinovosyldiacylglycerol synthase, *BTA* betaine lipid synthase, *PGPS* phosphatidylglycerophosphate synthase, *PGP* phosphatidylglycerophosphatase, *MIPS* myo-inositol-1-phosphate synthase, *PIS* phosphatidylinositol synthase, *SDC* serine decarboxylase, *ECT* CDP-Ethanolamine synthase, *EPT* ethanolamine phosphotransferase, *CHK* choline kinase, *LPCAT* lysophospholipid acyltransferases

Lipid metabolism consists of de novo fatty acid (FA) synthesis, FA elongation and desaturation, membrane lipid synthesis and turnover, TAG assembly and lipolysis, etc. [53]. According to Fig. 8, the genes involved in FA biosynthesis were transiently up-regulated after exposure to HL for 3 h and showed a down-regulation at 24 h; upon removal of the HL stress, many genes were recovered yet in a less extent. However, the transcript levels of genes responsible for de novo fatty acid biosynthesis were generally unchanged or down-regulated under phosphate starvation condition [54]. These genes were also strongly down-regulated upon exposure to nitrogen starvation condition [55]. The results suggest that the transcriptional down-regulation of de novo fatty acid biosynthetic genes is the common response of *P. tricornutum* to stresses. For the genes involved in the FA activation, acyl-CoA thioesterase (TE) and acyl-CoA-binding protein (ACBP) were up-regulated, long-chain acyl-CoA synthase (LACS2) was transiently up-regulated, while LACS3-5 were down-regulated upon HL (Fig. 8). HL induced the transcriptional up-regulation of many fatty acid desaturases (FADs) in a transient manner, including PAD (Phatr3_J9316), Δ12-FAD (Phatr3_J25769), Δ6-FAD (Phatr3_J29488), Δ5-FAD (Phatr3_J46830), Δ3-FAD (Phatr3_J5271), and Δ7-FAD (Phatr3_J28797) (Fig. 8). ω6-FAD (Phatr3_J48423) and several fatty acid elongases (FAEs) such as Δ6-FAE (Phatr3_J22274 and Phatr3_J20508) and Δ5-FAE (Phatr3_J34485), on the other hand, were down-regulated by HL (Fig. 8), indicating the important roles of them in EPA synthesis that was impaired under HL (Fig. 4). When the HL stress was removed, with several exceptions, the genes of de novo FA synthesis, FA activation, elongation and desaturation were generally recovered but slightly (Fig. 8).

In the process of TAG assembly, diacylglycerol acyltransferase (DGAT) catalyzes the final step and is critical for TAG biosynthesis [35]. There are four DGAT isoforms in *P. tricornutum*, one for type I (DGAT1) and 4 for type II (DGAT2A, DGAT2B, DGAT2C, and DGAT2D) [56]. Upon HL, DGAT1A, DGAT2B and DGAT2D were transcriptionally up-regulated (Fig. 8) and correlated with the increase of TAG (Fig. 3), supporting their role in TAG synthesis and consistent with our previous overexpression study that led to considerable TAG increase in *P. tricornutum* [56]. On the other hand, Scarsini et al. [55] reported two were slightly up-regulated (DGAT1 and DGAT2D) while the other two were down-regulated (DGAT2A and DGAT2C) in response to nitrogen starvation [55]. These results suggest the differential regulation of DGATs responding to diverse stresses. Other genes encoding enzymes in the Kennedy pathway, such as glycerol-3-phosphate acyltransferase (GPAT) and lysophosphatidate acyltransferase (LPAAT), showed differential expression patterns in response to HL: the chloroplastidic GPAT (Phatr3_J3262) was up-regulated while the extraplastidic one (Phatr3_J54709) was not affected; two LPAATs (Phatr3_J11916 and Phatr3_J20460) were transiently up-regulated and one was down-regulated (Fig. 8). It is worth noting that phospholipid: diacylglycerol acyltransferase (PDAT), which catalyzes the acyl-CoA-independent synthesis of TAG using membrane glycerolipids as acyl donors in *P. tricornutum* [57], was transcriptionally up-regulated throughout the whole HL treatment (Fig. 8). This result suggests that the enhancement of TAG under HL stress is predominantly due to the recycling of membrane phospholipids rather than de novo biosynthesis. The up-regulation of PDAT has also been observed in *P. tricornutum* during early and late phosphate starvation stress [54]. Furthermore, it has been reported by Yang et al. (2013) that nitrogen depletion leads to transcriptional up-regulation of PDAT and the remodeling of membranes in *P. tricornutum* [35]. All these results support that stress conditions cause the diversion of membrane phospholipids to TAG synthesis, and PDAT plays an important role in this process. Similarly, up-regulation of the stramenopile-type lipid droplet protein (StLDP), the most abundant and structural protein of the lipid droplet fraction [58], was also observed (Fig. 8). Nevertheless, when the HL stress was removed, the genes involve in TAG assembly didn’t or just recovered slightly at the transcriptional levels (Fig. 8).

Many genes involved in the synthesis of polar lipids were transcriptionally affected by HL in *P. tricornutum* (Fig. 8). Consistent with the decrease of MGDG (Fig. 3), monogalactosyldiacylglycerol synthase encoded genes (MGD; Phatr3_J14125, Phatr3_J54168 and Phatr3_J9619) were all down-regulated by the HL treatment (Fig. 8). Interestingly, digalactosyldiacylglycerol synthase (DGD) that catalyzes the synthesis of DGDG from MGDG, was transcriptionally up-regulated (Fig. 8), though DGDG declined in response to HL (Fig. 3). Probably, MGDG serves as the precursor of DGDG and its decrease contributes to DGDG decline. This is consistent with the results obtained under nitrogen deficiency conditions [55]. Nevertheless, both MGD and DGD were down-regulated upon phosphate stress [59]. Sulfoquinovosyldiacylglycerol synthase (SQD) is the key enzyme for SQDG synthesis. The SQD encoded genes (Phatr3_J21201 and Phatr3_J50356) were down-regulated by HL (Fig. 8), consistent with the decrease of SQDG in *P. tricornutum* (Fig. 3). Phosphatidylglycerophosphate synthase (PGPS) and phosphatidylglycerophosphatase (PGP), which are involved in PG synthesis, were transcriptionally down-regulated by HL as well (Fig. 8), yet PG level was not attenuated (Fig. 3). Besides, some enzymes such as choline kinase (CHK) and lysophospholipid acyltransferases (LPCAT) were even transcriptionally up-regulated by HL treatment (Fig. 8), probably providing PC for PDAT (utilizing PC as the acyl donor for TAG synthesis) to support the increase of TAG. It has been demonstrated that LPCAT utilizes the cytosolic acyl-CoA pool and lysophospholipids to regenerate PC and PE [60]. When the HL stress was removed, while many polar lipid synthetic genes did not or slightly recovered, several ones recovered considerably, including MDG (Phatr3_J54168), and PGPS (Fig. 8), indicative of the important role of these genes in polar lipid biosynthesis.

**Fig. 9:**
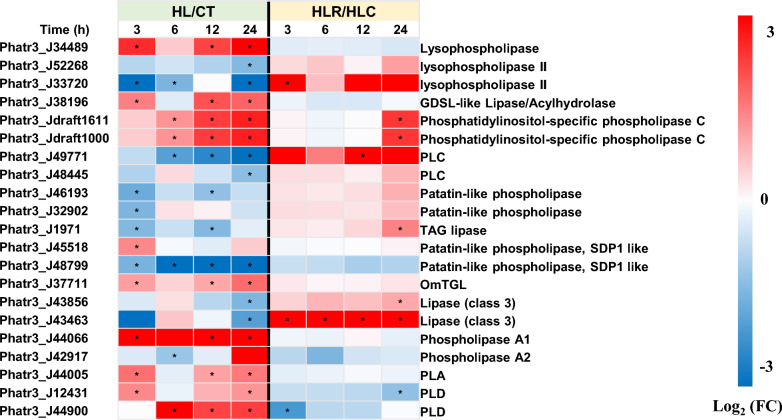
Differential expression of selected lipase genes in *P. tricornutum *during the two-stage treatment. *TGL* TAG lipase, *PL* phospholipase

There are a number of putative lipase-encoding genes found in *P. tricornutum* [61] and they showed differential responses to HL treatment (Fig. 9). A portion of lipase genes were considerably up-regulated, such as lysophospholipase (Phatr3_J34489), GDSL-like lipase/acylhydrolase (Phatr3_J38196), phosphatidylinositol-specific phospholipase C (Phatr3_Jdraft1611 and Phatr3_Jdraft1000), phosphatidic acid-preferring phospholipase A (PLA; Phatr3_J44005) and phospholipase D (PLD; Phatr3_J12431 and Phatr3_J44900). PLA hydrolyzes phospholipid substrates at specific ester bonds for releasing acyl groups, while PLD catalyzes hydrolysis of the phosphodiester bond of the glycerolipids to form PA [62]. The up-regulation of PLA and PLD enzymes may contribute to the recycling of phospholipids for TAG synthesis. When recovered from the HL stress, the up-regulation of these genes was abolished and even down-regulation started (Fig. 9). On the other hand, some lipases were transcriptionally down-regulated by HL treatment, for instance, lysophospholipase II (Phatr3_J52268 and Phatr3_J33720), Phospholipase C (PLC; Phatr3_J49771 and Phatr3_J48445), patatin-like phospholipase (Phatr3_J46193 and Phatr3_J32902) and TAG lipase (Phatr3_J1971). When recovered from the HL stress, lysophospholipase II, PLC and TAG lipase were reversibly up-regulated (Fig. 9). TAG lipase mediates the first initial step of TAG breakdown [61]. Degradation of TAG serves as energy resources, building blocks for lipid remodeling or membrane biosynthesis, and signaling molecules influencing gene transcription or enzyme activity [63, 64]. At the same time, the restoration of membrane lipids, in particular PG, DGTA and MGDG (Fig. 3A), as well as up-regulation of genes involved in glycolysis were observed when the HL stress was removed (Fig. 6).

### qPCR validation of selected RNA-seq data

**Fig. 10:**
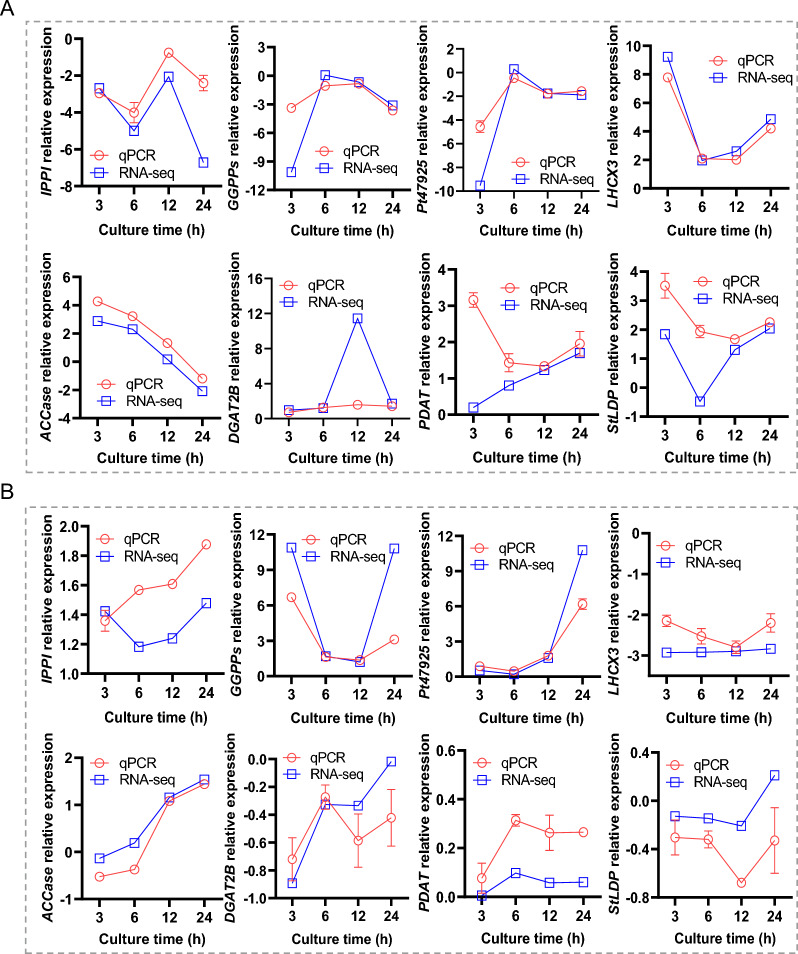
Time-resolved expression of selected genes determined by qPCR. **A** stage I (HL versus CT). **B** stage II (HLR versus HLC). The transcript level was expressed as log2 transformed value. The data are expressed as mean ± SD (*n* = 3)

To assess the expression pattern of DEGs from RNA-seq data, eight genes were selected for qPCR validation, including IPPI, GGPPS and Pt47925 that are involved in pigment metabolism, LHCX3 involved in photosynthesis, and ACCase, DGAT2B, PDAT and StLDP that are involved in lipid metabolism. Obviously, these genes showed strong responses to illumination transitions at the transcript level, and the qPCR results were generally consistent with the RNA-Seq data of stage I and stage II (Fig. 10A, B).

## Conclusion

*P. tricornutum* is cited as an emerging model of diatom for the production of a suite of natural and engineered products [4]. By integrating time-resolved physiochemical and transcriptomic data during HL and HLR stages, here we provided insights into the understanding of light-dependent responses of *P. tricornutum* during illumination transitions. Switching from HL to HLR stage caused the algal shift from one metabolic state, in which cells slowed down division and converted more carbon and energy to storage compounds particularly triacylglycerol, to another, in which the storage compounds were remobilized for cell regrowth. Besides, certain key enzymes involved in carotenoid biosynthesis and lipid metabolism of *P. tricornutum* were highlighted and putative monooxygenases that catalyze the ketolation step of fucoxanthin synthesis were proposed, which will aid in the future engineering of *P. tricornutum* for improved synthesis of target products.

## Supplementary Information

**Additional file 1: Fig. S1** Immunoblot analysis of photosynthetic proteins in *P. tricornutum* under various culture conditions. Cyt b6, cytochrome b6 protein, PsbD, D2 protein of PSII, LHCI, light harvesting complex of PSI. **Fig. S2** Correlation between fucoxanthin and chlorophyll *a* levels in *P. tricornutum* under different culture conditions. The data are from Fig. 2. **Fig. S3** FA relative abundance in lipids of day 1 and 2 cultures for CT, HL, HLC and HLR. **Fig. S4** Global analysis of transcriptomes and DEGs. (A) Principal component analysis (PCA) of the CT, HL, HLC and HLR transcriptomes. (B) Venn diagram illustrating the DEGs for HL versus CT and HLR versus HLC. (C) An overview of up and down DEGs for HL versus CT and HLR versus HLC. **Fig. S5** TLC plate picture of polar lipids.

**Additional file 2: Data S1** List of genes and their FPKM values of all samples.

**Additional file 3: Data S2** RNA-Seq data for the DEG pathway analysis.

**Additional file 4: Data S3** RNA-seq data of the hypothetical cytochrome P450 monooxygenase genes.

**Additional file 5: Table S1**. Primers used for qPCR validation of selected genes.

## Data Availability

All data generated or analyzed during this study are included in this published article and its supplementary information files.

## Abbreviations

AACT: Acetoacetyl-CoA thiolase
ACBP: Acyl-CoA-binding protein
ACH: Aconitate hydratase
ACS: Acetyl-CoA synthetase
ACL: ATP-citrate lyase
ACCase: Acetyl-CoA carboxylase
ACP: Acyl carrier protein
AK: Acetate kinase
ALAD: Amino levulinic acid dehydratase
ALDH: Aldehyde dehydrogenase
BGS: 1,3-Beta-glucan synthase
BGA: β-Glucanase
BTA: Betaine lipid synthase
CCDA1: Cyt c-type biogenesis factor
CCB1: Cyt c-type biogenesis factor
CHL: Mg-chelatase
CPOX: Coproporphyrinogen-III oxidase
CHLG: Chlorophyll synthetase
CS: Citrate synthase
CMK: 4-Diphosphocytidyl-2-C-methyl-D-erythritol kinase
CYP97A: Cytochrome P450 beta hydroxylase
CHK: Choline kinase
CRTISO: Carotenoid isomerase
DXS: 1-Deoxy-D-xylulose 5-phosphate synthase
DXR: 1-Deoxy-D-xylulose 5-phosphate reductoisomerase
DGD: Digalactosyldiacylglycerol synthase
DGAT: Diacylglycerol acyltransferase
ECT: CDP-Ethanolamine synthase
EPT: Ethanolamine phosphotransferase
ENO: Enolase
ELO: Fatty acid elongase
ENR: Enoyl-ACP reductase
Fd: Ferredoxin
FHD: Fumarate hydratase
FNR: Ferredoxin NADP reductase
FTRB: Ferredoxin-thioredoxin reductase
FCP: Fucoxanthin chlorophyll a/c protein
FBA: Fructose-bisphosphate aldolase
FBP: Fructose-1,6-bisphosphatase
FAD: Fatty acid desaturase
FAE: Fatty acid elongase
FPPS: Farnesyl diphosphate synthase
GTS: Glutamyl-tRNA synthetase
GTR: Glutamyl-tRNA reductase
GSA: Glutamate-semialdehyde aminotransferase
GUN4: Tetrapyrrole binding protein
GAPDH: Glyceraldehyde 3-phosphate dehydrogenase
GLK: Glucokinase, GPI glucose-6-phosphate isomerase
GPDH: Glycerol-3-phosphate dehydrogenase
G6PD: Glucose-6-phosphate 1-dehydrogenase
GPPS: Geranyl diphosphate synthase
GGPPS: Geranylgeranyl diphosphate synthase
GALE: UDP-galactose 4-epimerase
GPAT: Glycerol-3-phosphate acyltransferase
HCAR: 7-Hydroxymethyl chlorophyll a reductase
HDS: 4-Hydroxy-3-methylbut-2-en-1-yl diphosphate synthase
HCS: Hydroxymethylglutaryl-CoA synthase
HCR: HMG-CoA reductase
HAD: 3-Ketoacyl-ACP dehydratase
ISPD: 2-C-methyl-D-erythritol 4-phosphate cytidylyltransferase
ISPF: 2-C-methyl-D-erythritol 2,4-cyclodiphosphate synthase
IPPI: Isopentenyl-diphosphate Delta-isomerase
IDH: Isocitrate dehydrogenase
ISC1: Fe–S cluster assembly factor
KAS: 3-Ketoacyl-ACP synthase
KAR: 3-Ketoacyl-ACP reductase
LPCAT: Lysophospholipid acyltransferases
LPAAT: Lysophospholipid acyltransferases
LACS: Long-chain acyl-CoA synthetase
LCYb: Lycopene beta cyclase
LHC: Light harvest complex protein
MCT: Malonyl-CoA: acyl carrier protein transacylase
MDH: Malate dehydrogenase
MK: Mevalonate-5-kinase
MPK: Phosphomevalonate kinase
MPPD: Mevalonate-5-pyrophosphate decarboxylase
MDH: Malate dehydrogenase
ME: Malic enzyme
MGD: Monogalactosyldiacylglycerol synthase
MIPS: Myo-inositol-1-phosphate synthase
np-GAPDH: Glyceraldehyde 3-phosphate dehydrogenase (nonphosphorylating)
OGDH: 2-Oxoglutarate dehydrogenase
PPOX: Protoporphyrinogen IX oxidase
POR: Light-dependent protochlorophyllide oxidoreductase
PPH: Pheophytinase
PetC: Cytochrome b6-f complex iron–sulfur subunit
PsaO: Photosystem I subunit PsaO
PsbM: Photosystem II reaction center M protein
PsbW: Photosystem II PsbW protein
PsbO: Photosystem II oxygen-evolving enhancer protein 1
PsbP: Photosystem II oxygen-evolving enhancer protein 2
PsbQ: Photosystem II oxygen-evolving enhancer protein 3
Psb27: Photosystem II subunit 27
PsbU: Photosystem II extrinsic protein
PRK: Phosphoribulokinase
PEPC: Phosphoenolpyruvate carboxylase
PPDK: Pyruvate phosphate dikinase
PGM: Phosphoglucomutase
PFK: 6-Phosphofructokinase
PGK: Phosphoglycerate kinase
PGAM: Phosphoglycerate mutase
PK: Pyruvate kinase
PEPCK: Phosphoenolpyruvate carboxykinase
PYC: Pyruvate carboxylase
PDHC: Pyruvate dehydrogenase complex
PGLS: 6-Phosphogluconolactonase
PSY: Phytoene synthase
PDS: Phytoene desaturase
PAD: Palmitoyl-ACP delta-9-desaturase
PAP: Phosphatidate phosphatase
PDAT: Phospholipid: diacylglycerol acyltransferase
PL: Phospholipase
PGPS: Phosphatidylglycerophosphate synthase
PGP: Phosphatidylglycerophosphatase
PIS: Phosphatidylinositol synthase
RBCS: Ribulose-1,5-bisphosphate carboxylase small subunit
RPI: Ribose 5-phosphate isomerase
RPE: Ribulose-phosphate 3-epimerase
RPI: Ribose 5-phosphate isomerase
RPE: Ribulose-phosphate 3-epimerase
SCS: Succinyl-CoA synthetase
SDH: Succinate dehydrogenase
StLDP: Stramenopile-type lipid droplet protein
SQD: Sulfoquinovosyldiacylglycerol synthase
SDC: Serine decarboxylase
TE: Acyl-CoA thioesterase
TRK: Transketolase
TIM: Triosephosphate isomerase
TGL: TAG lipase
UROS: Uroporphyrinogen III synthase
UROD: Uroporphyrinogen III decarboxylase
UPP/UDP-glucose: Pyrophosphorylase
VDE: Violaxanthin de-epoxidase
VDL: Violaxanthin de-epoxidase like
VDR: Violaxanthin de-epoxidase-related
ZDS: Zeta-carotene desaturase
ZISO: Zeta-carotene isomerase
ZEP: Zeaxanthin epoxidase
6PGD: 6-Phosphogluconate dehydrogenase
