# Supplementary material for: Physiochemical and molecular responses of the diatom Phaeodactylum tricornutum to illumination transitions
Source: Biotechnol Biofuels Bioprod. 2023 Jun 16;16:103. doi: 10.1186/s13068-023-02352-w (PMC10276504; doi:10.1186/s13068-023-02352-w)
Supplement: Supplementary file 1 — Additional file 1: Fig. S1 Immunoblot analysis of photosynthetic proteins in P. tricornutum under various culture conditions. Cyt b6, cytochrome b6 protein, PsbD, D2 protein of PSII, LHCI, light harvesting complex of PSI. Fig. S2 Correlation between fucoxanthin and chlorophyll a levels in P. tricornutum under different culture conditions. The data are from Fig. 2. Fig. S3 FA relative abundance in lipids of day 1 and 2 cultures for CT, HL, HLC and HLR. Fig. S4 Global analysis of transcriptomes and DEGs. (A) Principal component analysis (PCA) of the CT, HL, HLC and HLR transcriptomes. (B) Venn diagram illustrating the DEGs for HL versus CT and HLR versus HLC. (C) An overview of up and down DEGs for HL versus CT and HLR versus HLC. Fig. S5 TLC plate picture of polar lipids. [file 13068_2023_2352_MOESM1_ESM.pdf]

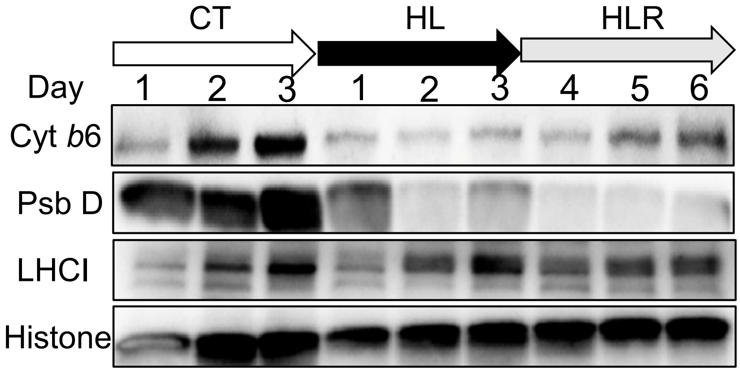

Fig. S1 Immunoblot analysis of photosynthetic proteins in *P. tricornutum* under various culture conditions. Cyt b6, cytochrome b6 protein, PsbD, D2 protein of PSII, LHCI, light harvesting complex of PSI.

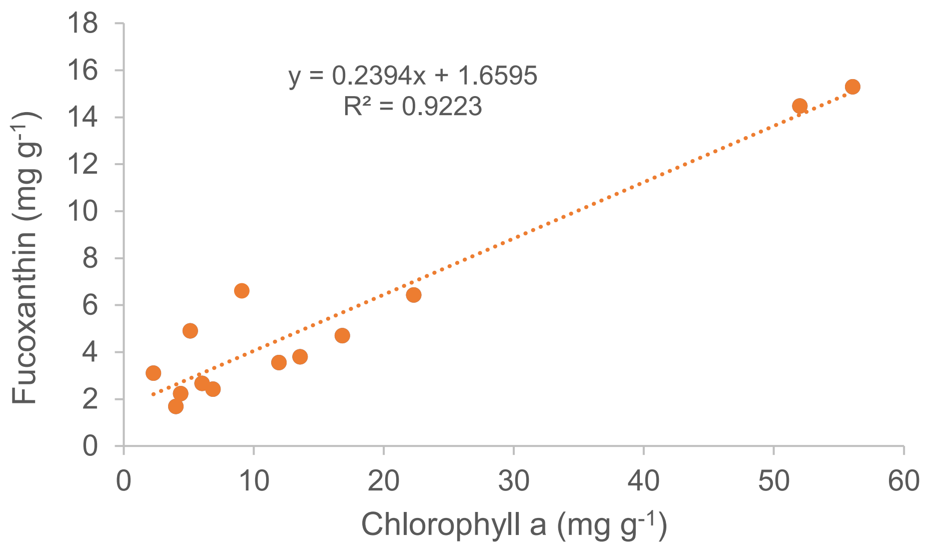

Fig. S2 Correlation between fucoxanthin and chlorophyll a levels in *P. tricornutum* under different culture conditions. The data were from Fig. 2.

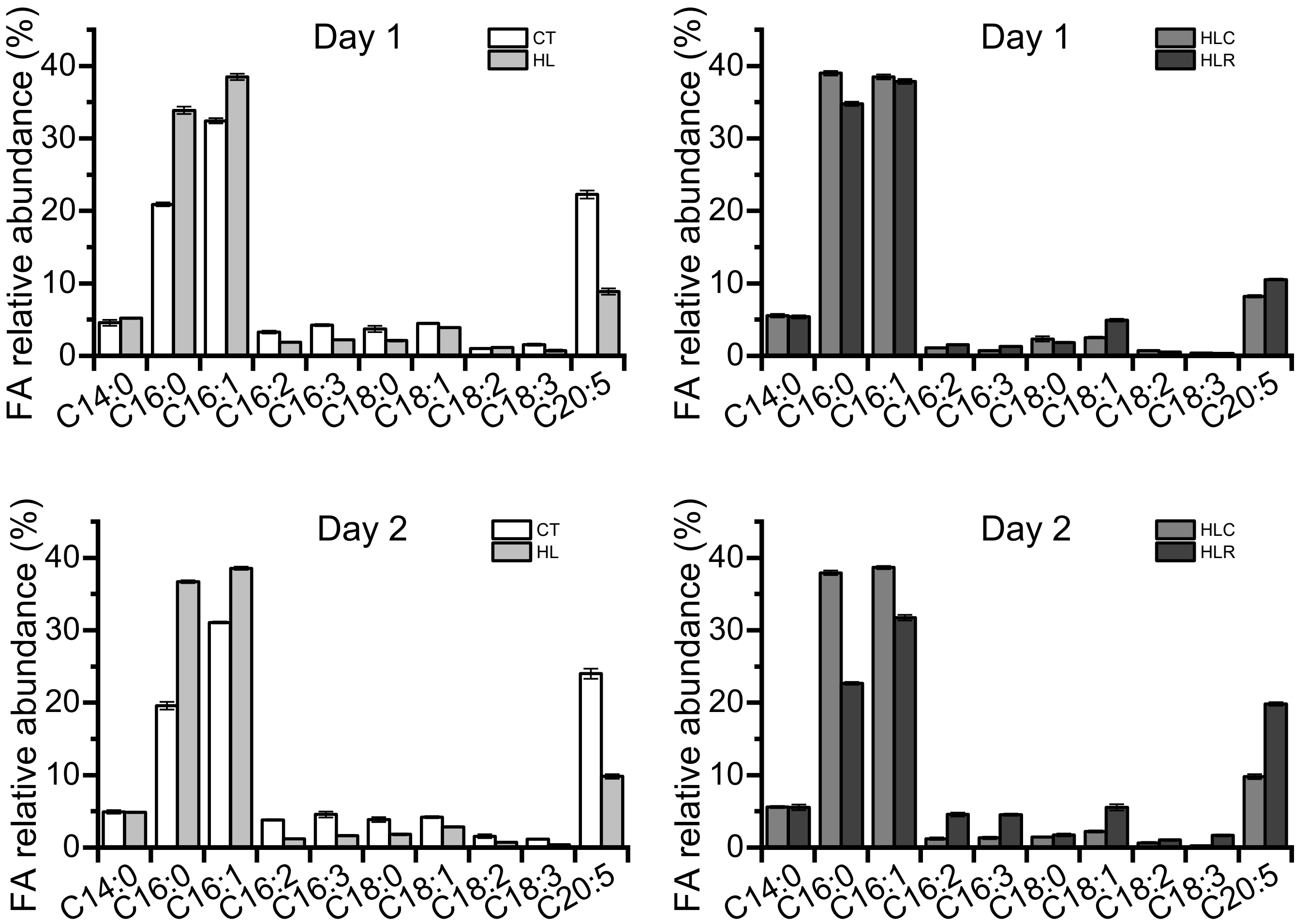

Fig. S3 FA relative abundance in lipids of day 1 and 2 cultures for CT, HL, HLC and HLR.

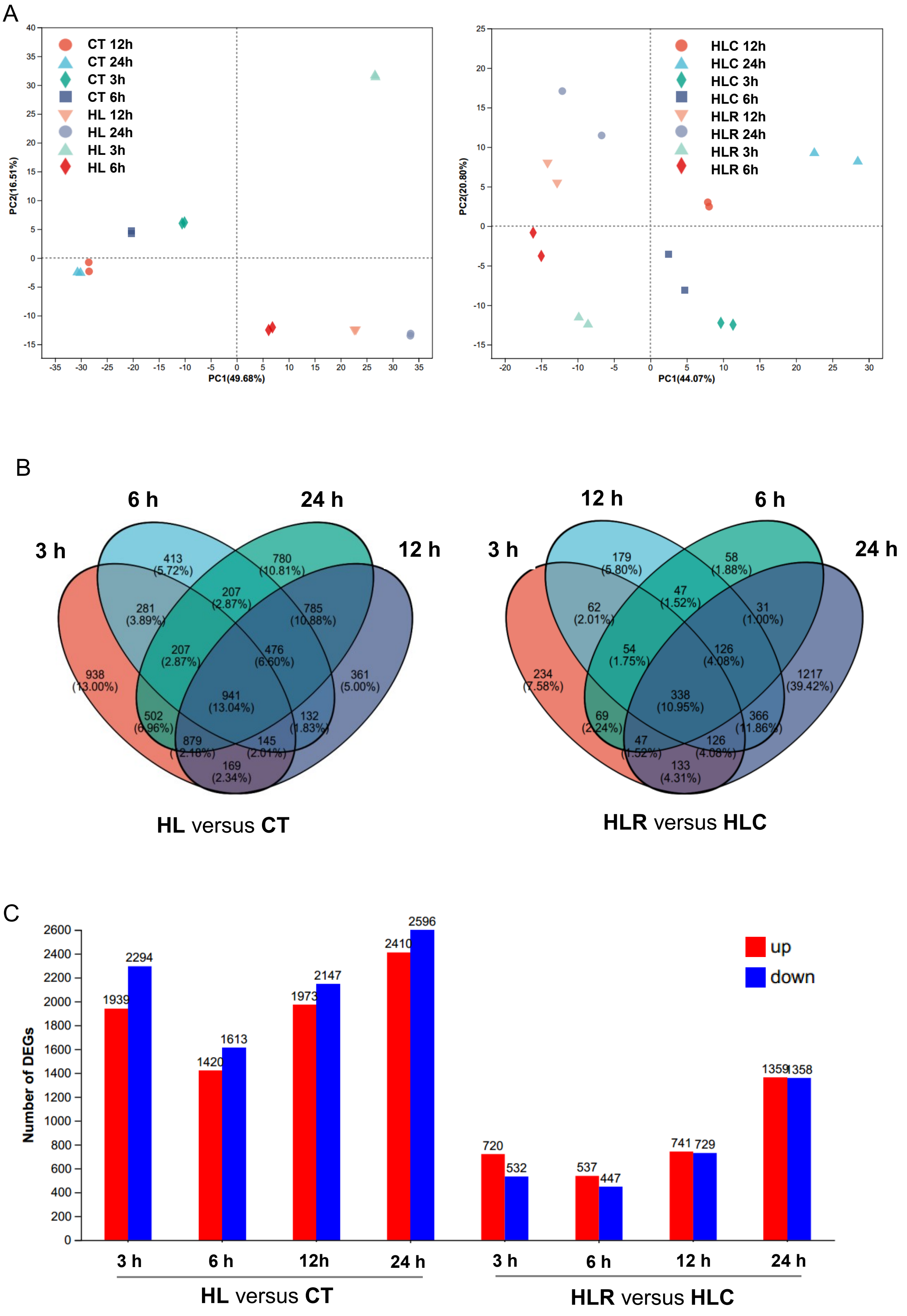

Fig. S4. Global analysis of transcriptomes and DEGs. (A) Principal component analysis (PCA) of the CT, HL, HLC and HLR transcriptomes. (B) Venn diagram illustrating the DEGs for HL versus CT and HLR versus HLC. (C) An overview of up and down DEGs for HL versus CT and HLR versus HLC.

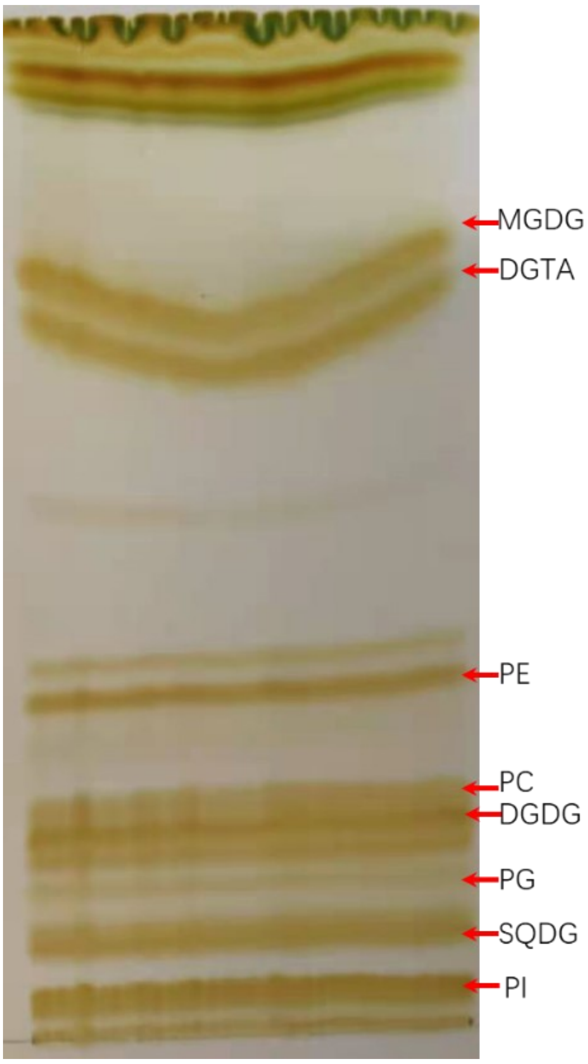

Fig S5. TLC plate picture of polar lipids
